# Triglyceride, fatty acid profile and antioxidant characteristics of low melting point fractions of Buffalo Milk fat

**DOI:** 10.1186/s12944-019-0995-6

**Published:** 2019-03-09

**Authors:** Imran Taj Khan, Muhammad Nadeem, Muhammad Imran, Muhammad Asif, Muhammad Kamran Khan, Ahmad Din, Rahman Ullah

**Affiliations:** 1 Department of Dairy Technology, University of Veterinary and Animal Sciences, Lahore, Punjab Pakistan; 2 Institute of Home and Food Sciences, Faculty of Life Sciences, Government College University, Faisalabad, Punjab Pakistan; 3 Planning and Development Division, Pakistan Agricultural Research Council, Islamabad, Pakistan; 4 Postharvest Research Center, Ayub Agricultural Research Institute, Faisalabad, Punjab Pakistan

**Keywords:** Low melting point fractions, Buffalo Milk fat, Triglyceride composition, Antioxidant capacity

## Abstract

**Background:**

Among the dietary lipids, milk fat is most complicated as it contains more than one hundred types of fatty acids and several triglycerides. Huge versatility in triglyceride and fatty composition makes it possible to convert milk fat into various fractions on the basis of melting characteristics. Functional properties of milk fat can be increased by converting it into different fractions. After cow milk, buffalo milk is the second largest source of milk and chemical characteristics of buffalo milk fat has been studied in a limited fashion. The main mandate was determination of triglyceride, fatty acid profile and antioxidant characteristics of low melting point fractions of buffalo milk fat for increased industrial applications.

**Methods:**

Buffalo milk fat (cream) was fractionated at three different temperatures i.e. 25, 15 and 10 °C by dry fractionation technique and packaged in 250 ml amber glass bottles and stored at ambient temperature for 90 days. The fraction of milk fat harvested at 25, 15 and 10 °C were declared as LMPF-25, LMPF-15 and LMPF-10. Unmodified milk fat was used as control (PBMF). Low melting point fractions were analyzed for triglyceride composition, fatty acid profile, total phenolic contents, DPPH free radicals scavenging activity, reducing power, free fatty acids, peroxide value, iodine value and conjugated dienes at 0, 45 and 90 days of storage.

**Results:**

In LMPF-10, concentrations of C_36_, C_38_, C_40_, and C_42_ were 2.58, 3.68, 6.49 and 3.85% lower than PBMF. In LMPF-25, concentrations of C_44_, C_46_, C_48_, C_50_, C_52_ and C_54_ were 0.71, 1.15, 2.53, 4.8, 0.39 and 2.39% higher than PBMF. In LMPF-15, concentrations of C_44_, C_46_, C_48_, C_50_, C_52_ and C_54_ were 2.45, 4.2, 3.47, 5.92, 2.38 and 3.16% higher than PBMF. In LMPF-10, concentrations of C_44_, C_46_, C_48_, C_50_, C_52_ and C_54_ were 2.8, 5.6, 5.37, 7.81, 3.81 and 4.45% higher than PBMF. LMPF-25, LMPF-15 and LMPF-10 had higher concentration of unsaturated fatty acids as compared PBMF. Total phenolic contents of buffalo milk fat and its fractions were in the order of LMPF-10 > LMPF-15, LMPF-25 > PBMF. Storage period of 45 days had a non-significant effect on total flavonoid content. 2, 2-Diphenyl-1-picrylhydrazyl free radical scavenging activity (DPPH) free radical scavenging activity of LMP-25, LMPF-15 and LMPF-10 were 4.8, 13.11 and 25.79% higher than PBMF. Reducing power of PBMF, LMPF-25, LMPF-15 and LMPF-10 were 22.81, 28.47, 37.51 and 48.14, respectively. Estimation of free fatty acids after the 90 days of storage duration, no significant difference was found in content of free fatty acids in unmodified milk fat and low melting point fractions. Testing of peroxide value in 90 days old samples showed that peroxide value of PBMF, LMPF-25, LMPF-15 and LMPF-10 was 0.54, 0.98, 1.46 and 2.22 (MeqO_2_/kg), respectively. Storage period up to 45 days had a non-significant effect on anisidine value, iodine value and conjugated dienes.

**Conclusion:**

Low melting point fractions of buffalo milk fat had higher concentration of unsaturated fatty acids and more antioxidant capacity than unmodified milk fat with reasonable storage stability.

## Background

Production of buffalo milk in the world is 90.3 million tons while in Pakistan average 31.252 million tons cow milk is produced. The share of buffalo milk produced in Pakistan to the world milk production is 62.8% [1]. Fat content of buffalo, cow and goat milk ranges from 6 to 7%, 4.2–4.5% and 4–4.2% [2]. The protein content of buffalo, cow and goat milk is 3.8–4%, 3.2–3.3% and 3.1–3.2%. The ash content of buffalo, cow and goat milk is 0. 82, 0.72 and 0.71% [3]. Viscosity of buffalo milk is 2.04 Cp as compared to 1.86 and 1.74 cp of cow and goat milk. Buffalo milk has low cholesterol 8 mg/100 g as compared to cow and goat milk which is 14 and 10 mg/100 g, respectively. Buffalo milk is healthier than cow milk in terms of lower concentration of cholesterol and higher magnitude of unsaturated fatty acids [4]. The lower cholesterol value of buffalo milk makes it more popular in the health-conscious market. The higher levels of the fats and proteins of buffalo milk makes it more economical alternative to cow milk for the production of casein, caseinates, whey protein concentrates and a wide range of the fat rich dairy products [5]. Concentration of vitamin E in buffalo and cow milk is 5.5 and 2.1 mg/ 100 ml, while the amount of vitamin C in buffalo and cow milk 3.66 and 0.94 mg/100 ml [5, 6]. Antioxidant activity of Vitamin E and Vitamin C is scientifically established [7]. Due to the difference in the concentration of antioxidant substances, buffalo and cow milks may have different antioxidant activity. Therefore, functional value of buffalo milk may be higher than cow milk. Increased incidences of metabolic diseases have led the consumers to choose make health choices of foods and demand for functional foods is mounting across the world. Changing life styles have led the food industry and researchers to develop functional foods and determine the functional value of traditional foods [8]. Milk fat is used in subcontinent since thousands of years as it is regarded as healthier fat than vegetable fats. Like other foods, researchers are also trying to enhance the functional value of milk fat and earlier investigations have shown that concentration of unsaturated fatty acids in low melting fractions of milk fat was more than parent milk fat [9]. Low melting fractions of milk fat are obtained by separating the fatty acids and triglycerides of higher melting point from the low melting point triglycerides by a suitable way and this process is known as fractionation. Fractionation may be performed by solvents or dry crystallization and dry crystallization is more suitable for industrial application due to no risk of solvent residues [10]. Milk fat is highly complex and composed of fatty acids, triglycerides of enormously different melting characteristics with large number of bioactive compounds and natural antioxidants [11]. Studies have shown that diets should contain enough concentration of antioxidants to avoid oxidative stresses, which lead to several diseases such as cancer, coronary heart diseases, accelerated ageing and breakdown of vital biochemical compounds [12]. The change in food consumption patterns have a great deal of impact on health, people in both developed and developing nations are facing problems of obesity, cancer, diabetes, allergies, ageing which are mostly due to the consumption of unbalanced diet. Nutraceuticals can functional foods should be used to decrease the disorders associated with unbalanced diet [13]. The melting characteristics of milk fat depend upon climatic conditions, breed, and stage of lactation and feed etc. [14]. Fractionation of milk fat can significantly alter the fatty acid composition of the fractions as compared to native milk fat [15]. The problem of lack of functional properties of milk fat, lower solid fat index and higher content of saturated fatty acids may be resolved by fractionating the milk fat into hard and soft fractions [16]. Stearin can be used as a bakery and confectionary fat due high melting point and solid fat index. Olein fractions may be used for cooking, frying and salad dressings etc. [17]. The acceptability of LMF for the health-conscious people is high [18]. Increased knowledge in free radical biology has led the consumer to consume those foods who has ample concentration of natural antioxidants. In such a situation, functional value of traditional foods should be discovered. Fatty acid and triglycerides profiles of low melting fractions of milk fat have been investigated and these findings have suggested that therapeutic value of low melting fractions of buffalo milk fat were greater than native milk fat, however, little is known regarding the antioxidant characteristics of low melting fractions of milk fat. No work has been done on the oxidative stability of LMF of buffalo milk fat. This investigation was designed to fractionate the buffalo milk fat at three different temperatures and study the oxidative stability of low melting point fractions at ambient temperature on the basis of certain chemical and oxidative stability characteristics.

## Methods

### Raw materials and preparation of Milk fat

Buffalo milk was obtained from Dairy Animals Training and Research Centre, University of Veterinary and Animal Sciences Lahore. All the chemicals used in this study were HPLC grade and obtained from Sigma Chemical Co. (St. Louis, MO). For the preparation of milk fat, fat was removed from buffalo milk by cream separator. Uncultured and unsalted butter was prepared from buffalo milk, which were then converted to milk fat.

### Preparation of low melting fractionation of Buffalo Milk fat

Buffalo milk fat was heated in 1-l beakers to 70 °C for 15-min in the water bath, transferred to a thermostatically controlled water bath at slowly cooled down 10 °C (1 °C/minute) held at 25, 15 and10°C for 2.5-h, low melting point fractions of buffalo milk fat were separated by vacuum filtration (600-mmHg pressure) on Buckner filtration assembly. The fraction of milk fat harvested at 25, 15 and 10^o^C were declared as LMPF-25, LMPF-15 and LMPF-10. Unmodified milk fat was used as control (PBMF). Each fractionation practice was repeated six times to minimize the variation [19]. The low melting point fractions were packaged in 250 ml amber glass bottles and stored at room temperature, oxidative stability was measured for a period of 90-days at the interval of 45-days.

### Chemical characteristics of low melting point fractions of Buffalo Milk fat

Low melting fractions of buffalo milk fat were characterized for free fatty acids, color, unsaponifiable matter, saponification value, iodine value, refractive index and peroxide value according to the standard methods [20].

### Antioxidant characteristics of low melting point fractions of Buffalo Milk fat

Following assays were performed to determine the antioxidant characteristics of LPMF, these antioxidant assays were performed at 0, 45 and 90 days of storage period.

### Total phenolic contents

Total phenolic contents in low melting fractions of milk fat were determined according to the method prescribed by Singleton et al. [21] with Gallic acid as standard. 100 μL sample was mixed with 100 μL methanol, 700 μL sodium carbonate was added and vortexed at 200 rpm. Contents of the test tube were incubated in the dark for 20 min, absorbance was measured at 735 nm on a spectrophotometer (Stalwart, USA). Total phenolic contents were calculated from calibration curve and reported as mg GAE/ml.

### Total flavonoids

Total flavonoid content of low melting fractions of milk fat was determined by the method prescribed by Nile and Khobragade [22]. Sample (0.5 ml) was mixed with 0.5 ml AlCl_3_ (2% in methanol), test tubes were incubated at room temperature of 1 h, absorbance was measured on a spectrophotometer at 420 nm, total flavonoid content were determined using Rutin as internal standard and reported as Quercetin equivalent (mg/g).

### 2, 2-Diphenyl-1-picrylhydrazyl (DPPH) assay

DPPH free radical scavenging activity of the low melting point fractions of milk fat was estimated by the method of Sanchez-Moreno [23]. 0.1 mM solution of DPPH was prepared in methanol, 100 μL sample was mixed with 2.9 ml DPPH and vortexed at 2200 rpm, followed by the incubation of tubes at room temperature for 30 min. Absorbance was recorded at 517 nm in visible region of spectrum using methanol as blank.

### Reducing power of low melting point fractions

Reducing power of low melting fractions of milk fat was determined according to the method prescribed by Adesegun et al. [24]. Briefly, 2.5 ml sample was mixed with 2.5 ml solution of potassium ferricyanide (1%), test tubes were incubated at 50oC for 20 min. 2.5 ml trichloroacetic acid (10%) was added and tubes were centrifuged at 1000 x g for 10 min. The supernatant 2.5 ml was mixed with 2.5 ml distilled water and 0.5 ml ferric chloride (0.1%). Absorbance was recorded on a double beam spectrophotometer at 700 nm (Stalwart, USA).

### α-Tocopherol

α-Tocopherol in low melting point fractions of milk fat was determined by the method of Jang and Xu [25]. 0.2 g sample was mixed with 2 ml HPLC grade n-hexane (Sigma Aldrich, USA), contents of test tube were vortex at 200 rpm for 30 s and 25 μL was injected into HPLC (Miford, MA; 715 Ultra WISP injector; 25 cm × 4.6 mm diameter 5-μm Supelcosil LC-Si (Supelco, Bellefonte, PA). Mobile phase was comprised of 0.5% ethyl acetate and 0.5% acetic acid in hexane, flow rate was adjusted at 1.5 ml/ min.

### Determination of vitamin a

Milk sample (20 ml) was taken in test tube for detecting vitamin A quantification. Ammonia with concentration of 5 ml and ethanol 20 ml was added to the sample. After mixing the solution it was kept for 10 min, supernatant was extracted and BHT (0.0025%) was added to it. Rotary evaporator was used for evaporation of solvent at 35 °C. 30 ml potassium hydroxide (5% in ethanol) was added for saponification and n-hexane was used for extraction. Again, the rotary evaporator was used for evaporation. 20 μl was taken and injected into HPLC which has RP-18 column, waters 990 detector and pumping system of LC-20 AT. Mobile phase was made of Acetonitril-methanol 85:15 in isocratic system [26].

### Triglyceride profile

For determination of triglyceride profile in milk, 50 mg of milk fat was taken in test tube and 1.0 mL n-hexane was added for dissolution. From this mixture, 1 μL was taken and directly injected into GC (7890-B, Agilent Technologies), fitted with a methyl lignoserate-coated (film thickness 0.25 l m), SP-2330 (SUPELCO Inc. Supelco Park Bellefonte, PA 16823–0048, USA) polar capillary column (RTX 65-TG) using mass spectrophotometer. The temperature of injector was 60 °C for 12 s and followed by increasing from 99 °C/min to 370 °C for holding time of 5 min. Oven temperature from 250 °C for about 2 min followed by an increase to 360 °C at 5 °C/min with holding time of 4 min. 370 °C temperature was set for detector and hydrogen was used as carrier gas with flow rate of 1.5 mL/min The split ratio was 1:  80 [27].

### Fatty acid profile

Fatty acids profile was determined as fatty acid methyl esters, 300-μL melted and well mixed sample was taken into 11-mL screw capped test tube, dissolved in 3-mL isooctane and 2-mL 0.5 N sodium methoxide was added vortexed at 1500 rpm for 3-min, allowed to separate for five minutes and the supernatant was injected into GC-MS (7890-B, Agilent Technologies), fitted with a methyl lignoserate-coated (film thickness 0.25 l m), SP-2560 column (100 m × 0.32 mm) using flame ionization detector as per standard IUPAC method [28]. Temperature of the inlet was set at 200 °C, temperature of FID was set at 250 °C, helium, hydrogen and oxygen were used at the rate of 2 ml/min, 4 ml/min and 40 ml/min.

### Lipid oxidation

For the estimation of lipid oxidation in low melting point fractions of buffalo milk fat, free fatty acids, peroxide value, iodine value and anisidine values were determined [20]. Conjugated dienes were estimated according to the methods [29].

### Statistical analysis

Triplicate samples of each olein fraction/ storage period were taken. Every sample was analyzed three times and the data was expressed as mean (*n* = 3 × 3; ±SD n = 3 × 3). Data were analyzed by using two-way analysis of variance techniques to find out the effect of treatment and storage by using SAS 9.1 (Statistical Analysis Software) software. *P*-value of 0.05 and 0.01 was used to denote the significant and highly significant difference.

## Results and discussion

### Chemical characteristics of low melting point fractions of Milk fat

Chemical and physical characteristics of different fractions of buffalo milk fat are mentioned in Table 1. Free fatty acids, peroxide value and color of LMPF-25, LMPF-15 and LMPF-10 were not different from the PBMF. LMPF-15 and LMPF-10 had higher magnitude of unsaponifiable matter (*p* < 0.05). Cholesterol and vitamins belong to unsaponifiable fraction, higher amount of unsaponifiable matter in the low melting point fractions can be justified by the affiliation of cholesterol and vitamins to the low melting point fractions. Nadeem et al. [9] studied the chemical characteristics of low melting point fractions of milk fat, concentration of cholesterol and unsaponifiable matter were higher in the low melting point fractions. Cheddar cheese prepared from low melting point fractions of milk fat had higher content of cholesterol than parent milk fat [30]. Refractive index and iodine value of low melting point fractions were higher than native milk fat. Iodine values of PBMF, LMPF-25, LMPF-15 and LMPF-10 were 34.22, 37.62, 41.18 and 46.77 cg/100 g, respectively. Iodine value and refractive index are associated with the degree of unsaturation present in oils and fats. Higher the unsaturation, more would be iodine value and refractive index [31]. Iodine value of milk fat was 33.8 [32]. Slip melting point was inversely related with iodine value. Slip melting point of PBMF, LMPF-25, LMPF-15 and LMPF-10 were 34.2, 25.8, 16.2 and 10.6 °C. Color of all the low melting point fractions of buffalo milk fat were not different from each other. Chemical characteristics of high oleic fraction of *Moringa oleifera* oil were different from the parent oil [33]. Azeem et al. [34] compared the chemical characteristics of fractionated cottonseed oil with unmodified oil, refractive index and iodine value oil winterized cottonseed oil and native oil were significantly different [34].

**Table 1:** Chemical characteristics of low melting point fractions of buffalo milk fat

Parameter	PBMF	LMPF-25	LMPF-15	LMPF-10
Free Fatty Acids%	0.11 ± 0.01^a^	0.12 ± 0.02^a^	0.11 ± 0.01^a^	0.10 ± 0.01^a^
USM	0.62 ± 0.03^c^	0.66 ± 0.04^c^	0.78 ± 0.07^b^	0.89 ± 0.09^a^
Refractive Index	1.451 ± 0.01^b^	1.453 ± 0.02^b^	1.457 ± 0.04^a^	1.459 ± 0.03^a^
Iodine Value	34.22 ± 1.21^d^	37.62 ± 1.89^c^	41.18 ± 2.55^b^	46.77 ± 2.61^e^
Slip Melting Point ^o^C	34.2 ± 0.31^a^	25.8 ± 0.24^b^	16.2 ± 0.45^c^	10.6 ± 0.50^d^
Peroxide Value	0.22 ± 0.03^a^	0.25 ± 0.02^a^	0.27 ± 0.01^a^	0.22 ± 0.04^a^
Cholesterol mg/100 g	165 ± 2.11^d^	181 ± 2.56^c^	216 ± 3.65^b^	235 ± 4.15^a^
Color*	R2.8 + 28Y^a^	R2.7 + 28Y^a^	R2.7 + 27Y^a^	R2.8 + 30Y^a^

In one row, if means are expressed with a different letter, these are statistically significant (*P* < 0.05)

PBMF: Parent Buffalo Milk Fat

LMPF-25: Low Melting Point Fraction of Buffalo Milk Fat Obtained at 25 °C

LMPF-15: Low Melting Point Fraction of Buffalo Milk Fat Obtained at 15 °C

LMPF-10: Low Melting Point Fraction of Buffalo Milk Fat Obtained at 10 °C

### Triglyceride profile

**Fig. 1:**
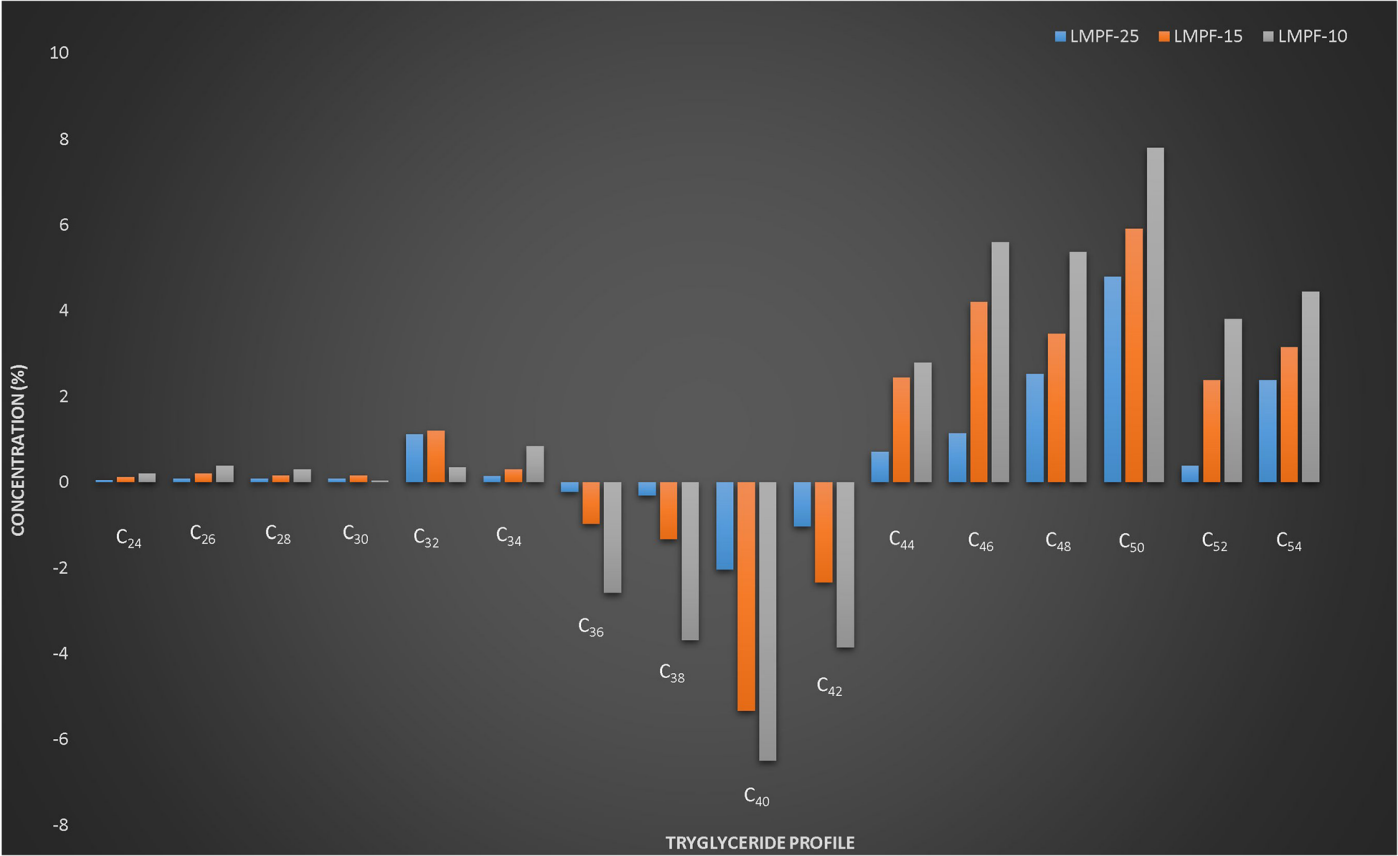
Triglyceride Profile of Low Melting Point Fractions of Buffalo Milk Fat

Buffalo milk contains about 6–7% fat, major portion (approximately 98%) is comprised of triglycerides present in fat globules [35]. Several factors affect the lipid composition such as stage of lactation, fat content and seasons [36]. However, the effect of fractionation temperature on triglyceride profile has been studied in a limited way. Triglyceride profile of milk has a great importance from physicochemical and functionality viewpoints [37]. These characteristics are important from the processing and value addition of milk and dairy products [38]. Table 2 presents the results of triglyceride profile of milk fat fractions obtained at 25, 15 and 10 °C. Triglyceride profile of unmodified milk fat was not different from the literature [39]. Fractionation temperature had a significant effect on triglyceride profile. Number of short-chain triglycerides (C_24_-C_34_) in low melting point fractions of buffalo milk fat were more than unmodified milk fat. In LMPF-25, concentrations of C_24_, C_26_, C_28_, C_30_, C_32_ and C_34_ were 0.05, 0.09, 0.09, 0.08, 1.12 and 0.15% higher than PBMF. In LMPF-15, concentrations of C_24_, C_26_, C_28_, C_30_, C_32_ and C_34_ were 0.12, 0.20, 0.16, 0.16%, 1.21 and 0.30% higher than PBMF. In LMPF-10, concentrations of C_24_, C_26_, C_28_, C_30_, C_32_ and C_34_ were 0.20, 0.39, 0.30, 0.31, 0.35 and 0.84% higher than PBMF. In LMPF-25, concentrations of C_36_, C_38_, C_40_, and C_42_ were 0.23, 0.31, 2.04 and 1.03% lower than PBMF. In LMPF-15, concentrations of C_36_, C_38_, C_40_, and C_42_ were 0.98, 1.33, 5.33 and 2.33% lower than PBMF (Fig. 1). Observable similarities studied by Gunstone [40] showed that triglyceride profile of milk fat was dependent upon the fractionation temperature. Jeyarani et al. [41] also suggested that triglyceride composition of the low melting point fractions was different form the normal milk fat.

**Table 2:** Triglyceride profile of low melting point fractions of buffalo milk fat (mg/100 g)

TAG Carbon Number	PBMF	LMPF-25	LMPF-15	LMPF-10
C_24_	0.07 ± 0.01^d^	0.12 ± 0.01^c^	0.19 ± 0.03^b^	0.27 ± 0.01^a^
C_26_	0.52 ± 0.02^d^	0.61 ± 0.03^c^	0.72 ± 0.09^b^	0.81 ± 0.12^a^
C_28_	0.44 ± 0.05^d^	0.53 ± 0.04^c^	0.60 ± 0.08^b^	0.74 ± 0.02^a^
C_30_	0.81 ± 0.01^d^	0.89 ± 0.05^c^	0.97 ± 0.04^b^	1.12 ± 0.05^a^
C_32_	1.17 ± 0.09^d^	1.29 ± 0.08^c^	1.38 ± 0.13^b^	1.52 ± 0.13^a^
C_34_	2.62 ± 0.06^d^	2.77 ± 0.11^c^	2.92 ± 0.06^b^	3.44 ± 0.17^a^
C_36_	6.75 ± 0.19^a^	6.52 ± 0.18^b^	5.77 ± 0.08^c^	4.17 ± 0.14^d^
C_38_	11.66 ± 0.15^a^	11.64 ± 0.27^b^	10.33 ± 0.19^c^	8.15 ± 0.12^d^
C_40_	14.47 ± 0.21^a^	10.43 ± 0.21^b^	9.14 ± 0.26^c^	7.98 ± 0.05^d^
C_42_	8.21 ± 0.34^a^	7.18 ± 0.31^b^	5.88 ± 0.26^c^	4.36 ± 0.14^d^
C_44_	5.43 ± 0.28^d^	6.14 ± 0.16^c^	7.88 ± 0.16^b^	8.23 ± 0.19^a^
C_46_	5.22 ± 0.25^d^	6.37 ± 0.09^c^	9.42 ± 0.28^b^	10.82 ± 0.42^a^
C_48_	6.92 ± 0.44^d^	9.45 ± 0.14^c^	10.39 ± 0.33^b^	12.29 ± 0.55^a^
C_50_	8.74 ± 0.16^d^	13.54 ± 0.17^c^	14.66 ± 0.74^b^	16.55 ± 0.73^a^
C_52_	11.81 ± 0.26^d^	12.20 ± 0.21^c^	14.19 ± 0.91^b^	15.62 ± 0.31^a^
C_54_	4.98 ± 0.33^d^	7.33 ± 0.28^c^	8.14 ± 0.18^b^	9.43 ± 0.24^a^

Within one row, if means are expressed with different letter these are statistically non-significant (*p* < 0.05)

PBMF: Parent Buffalo Milk Fat

LMPF-25: Low Melting Point Fraction of Buffalo Milk Fat Obtained at 25 °C

LMPF-15: Low Melting Point Fraction of Buffalo Milk Fat Obtained at 15 °C

LMPF-10: Low Melting Point Fraction of Buffalo Milk Fat Obtained at 10 °C

### Fatty acid profile

Fatty acid profile of unmodified milk fat and different fractions is mentioned in Table 3. Major changes were observed around medium and long chain fatty acids. In the fraction obtained at 10 °C, the average concentration of medium chain fatty acids was decreased from 48.79 to 36.71% which was 24.76% less than PMF. Concentration of stearic acid was steadily decreased from PMF to lower melting point fractions. Another markedly significant change was recorded in the extent of C_18:1_ and C_18:2_ which were increased by 7.73, 14.26 and 28.94% at fractionation temperatures of 25, 15 and 10 °C, respectively. Foods rich in monounsaturated fatty acids are becoming more popular owing to many medical benefits and extended storage capabilities [42]. They also possess the capability of have an impact on the lowering of blood cholesterol [43]. The fatty acid composition of milk fat fractions obtained at different temperature by dry crystallizations technique was significantly different from the native milk fat Van Aken et al. [15]. Major change in the fatty acid composition of milk fat fractions were also observed by Chen et al. [44]. Functional properties of milk fractions obtained by different techniques were appreciably different from the PMF [45]. Conjugated linoleic acid is a combination of several positional and geometric isomers of octadecadienoic acid having two conjugated double bonds. The double bond in the chain may be in cis or *trans* form, chains having trans double bond have therapeutic activity and are bioactive [46]. Earlier investigations have shown that trans fatty acids produced in partial hydrogenation and deodorization of vegetable oils and fats are harmful, while the trans fatty acid produced in rumen as a result of bio-hydrogenation are not harmful [47]. More than 12 isomers of conjugated linoleic acid have been discovered in meat and dairy products. Among the several isomers of conjugated linoleic acid, c-9, t-11 and t-10, c-12 has great physiological significance such as anti-diabetic, anti-carcinogenic and anti-atherosclerotic effects. Their therapeutic role as immunity booster has also been recognized [48]. IP et al. [49] fractionated the milk fat into various fractions and observed that the concentration of linoleic acid was significantly higher in the low melting point fraction collected at 10 °C. LMF were superior to parent milk fat for having 28% more vaccenic acid [50]. Magnitude of conjugated linoleic acid in buffalo milk was 4.83 mg/g. Strong correlation was found between conjugated linoleic acid and vaccenic acid [51]. Amount of conjugated linoleic acid in buffalo milk-based yoghurt was 6.1 mg/g [52].

**Table 3:** Fatty acid profile of low melting fractions of buffalo milk fat (mg/100 g)

Fatty Acids	PBMF	LMPF-25	LMPF-15	LMPF-10
C_4:0_	3.75 ± 0.04^b^	4.13 ± 0.11^a^	4.35 ± 0.16^a^	4.52 ± 0.05^a^
C_6:0_	2.28 ± 0.07^a^	2.43 ± 0.03^a^	2.55 ± 0.08^a^	2.71 ± 0.12^a^
C_8:0_	1.33 ± 0.02^a^	1.35 ± 0.04^a^	1.41 ± 0.06^a^	1.54 ± 0.02^a^
C_10:0_	3.42 ± 0.03^c^	2.95 ± 0.08^c^	3.24 ± 0.03^b^	4.10 ± 0.04^a^
C_12:0_	4.25 ± 0.06^b^	4.44 ± 0.14^b^	4.65 ± 0.05^a^	5.08 ± 0.02^a^
C_14:0_	11.68 ± 0.29^a^	11.45 ± 0.19^a^	11.33 ± 0.17^a^	11.18 ± 0.32^a^
C_16:0_	32.86 ± 0.67^a^	32.17 ± 0.35^a^	27.12 ± 0.44^b^	20.45 ± 0.25^c^
C_18:0_	9.35 ± 0.38^a^	8.92 ± 0.21^b^	7.95 ± 0.31^c^	5.85 ± 0.19^d^
C_18:1_	21.18 ± 0.97^d^	22.68 ± 0.34^c^	24.35 ± 0.54^b^	29.64 ± 0.49^a^

Within the row means with different letter are different (*P* < 0.05)

PBMF: Parent Buffalo Milk Fat

LMPF-25: Low Melting Point Fraction of Buffalo Milk Fat Obtained at 25 °C

LMPF-15: Low Melting Point Fraction of Buffalo Milk Fat Obtained at 15 °C

LMPF-10: Low Melting Point Fraction of Buffalo Milk Fat Obtained at 10 °C

### Antioxidant characteristics

Natural antioxidants slake the dreaded free radicals which are responsible for physiological stress, ageing, cancer and atherosclerosis [53, 54]. Scientists believe that antioxidants rich foods may provide protection against cardiovascular diseases, mutilation of nucleic acids and other deteriorating processes [55]. Most of the natural antioxidants are present in foods of plant origin. Phytochemicals can perform as reducing agents, metal binders, quenchers of singlet and triplet oxygen and reducing agents [56]. Milk fat also contains fat soluble vitamins, selenium and carotene in smaller amounts. Therefore, milk and dairy products are not significant sources of antioxidants. In this investigations, low melting point fractions of milk fat showed higher antioxidant activity than parent milk fat. In subcontinent, milk fat has several food applications, it is used in the preparation of delicious traditional sweets. On an average basis, milk fat contains about 70% saturated fatty acids [57]. Nutritionist and dieticians suggest to decrease the amount of saturated fatty acids. For the characterization of antioxidant capacity of low melting point fractions of buffalo milk fat, total phenolic contents, total flavonoids content, DPPH free radicals scavenging activity and reducing power were estimated. Phenolic compounds are excreted in milk from the forages or feed given to bovines [58]. Several studies have confirmed the antioxidant activity of phenolic compounds in food systems [59]. Total phenolic content of LMPF-10 were 1.90 mg GAE/g higher than PBMF (Table 4). Total phenolic contents of buffalo milk fat and its fractions were in the order of LMPF-10 > LMPF-15, LMPF-25 > PBMF. Total phenolic contents were not affected by the storage days up to 45 days in PBMF and all the three low melting fractions. Analysis of PBMF and its low melting fractions after 90 days of storage indicated that total phenolic contents decreased from its initial value. The decline in the amount of total phenolic contents in PBMF, LMFP-25, LMFP-15 and LMFP-10 were 1.31, 1.24, 0.74 and 1.02 mgGAE/g, respectively. The lesser decline of total phenolic contents in low melting point fraction as compared to the PBMF can be justified by the existence of higher concentration of natural antioxidant substances in low melting point fractions as compared to PBMF. Total phenolic contents of low melting point milk fat decreased during the long-term storage [60]. Storage period significantly affected the total phenolic contents of milk fat [57]. Total flavonoid content of all the three low melting point factions of milk fat were more than PBMF and were in the range of LMPF-10 > LMPF-15 > LMPF-25 > PBMF. Storage period of 45 days had a non-significant effect on total flavonoid content. For the characterization of antioxidant capacity of natural antioxidants, DPPH free radicals scavenging assay is universally used [8]. For the characterization of antioxidant capacity of blends of butter oil and high oleic acid fraction of *Moringa oleifera* oil, Nadeem et al. [33] used DPPH free radical scavenging activity as a marker of antioxidant capacity. DPPH free radical scavenging activity of butter oil decreased during the storage of 3 months [57]. Fractionation temperature considerably influenced the DPPH free radical scavenging activity. DPPH free radical scavenging of all the low melting point fractions of was higher than PBMF. Low melting fractions collected at lower temperature had higher DPPH free radical scavenging activity. DPPH free radical scavenging activity of LMP-25, LMPF-15 and LMPF-10 were 4.8, 13.11 and 25.79% higher than PBMF. Storage temperature up to 45 days had a non-significant effect on DPPH free radical scavenging activity. After 90 days of storage, the drop in DPPH free radical scavenging activity of PBMF, LMPF-25, LMPF-15 and LMPF-10 were 6.68, 5.77, 4.73 and 2.91%. Fazal et al. [61] compared the antioxidant characteristics of high oleic acid fraction of *Moringa oleifera* oil with soybean and sunflower oil, high oleic acid fraction of *Moringa oleifera* oil had more antioxidant capacity than unmodified soybean and sunflower oils. Fractionation temperature significantly affected the reducing power, the lower the fraction temperature, more was the reducing powder. Reducing power of PBMF, LMPF-25, LMPF-15 and LMPF-10 were 22.81, 28.47, 37.51 and 48.14. Reducing power of low melting point factions of milk fat reported in this investigation were higher than whole cow and buffalo milk as reported by Khan et al. [58]. Storage duration of 45 days had a non-significant effect on reducing power of all the low melting point fractions. However, reducing power of PBMF was significantly less than the initial value. Reducing power activity of yogurt prepared from camel milk was less than parent milk in storage period of 21 days [62].

**Table 4:** Antioxidant characteristics of low melting fractions of buffalo milk fat

Parameter	Storage Days	PBMF	LMPF-25	LMPF-15	LMPF-10
Total Phenolic Contents (mg GAE/g)	0	5.29 ± 0.14^f^	5.88 ± 0.17^e^	6.46 ± 0.11^b^	7.19 ± 0.08^a^
	45	4.21 ± 0.07^f^	5.79 ± 0.12^e^	6.39 ± 0.09^b^	7.11 ± 0.21^a^
	90	3.98 ± 0.10^h^	4.55 ± 0.15^g^	5.72 ± 0.18^d^	6.17 ± 0.13^c^
Total Flavonoids (mg Quercetin/g)	0	0.12 ± 0.01^f^	0.17 ± 0.03^e^	0.29 ± 0.02^c^	0.46 ± 0.05^a^
	45	0.10 ± 0.01^f^	0.15 ± 0.04^e^	0.27 ± 0.06^c^	0.41 ± 0.07^a^
	90	0.07 ± 0.02^g^	0.11 ± 0.01^f^	0.22 ± 0.03^d^	0.37 ± 0.02^b^
DPPH Free Radicals Scavenging Activity (%)	0	38.51 ± 0.82^g^	43.31 ± 1.12^f^	51.62 ± 0.76^c^	64.30 ± 1.35^a^
	45	38.42 ± 0.94^g^	42.64 ± 0.57^f^	50.27 ± 1.64^d^	63.22 ± 1.16^a^
	90	31.83 ± 1.55^i^	37.54 ± 0.66^h^	46.89 ± 1.34^e^	61.39 ± 1.29^b^
Reducing Power	0	22.81 ± 0.73^d^	28.47 ± 0.95^c^	37.51 ± 1.19^b^	48.14 ± 1.38^a^
	45	22.69 ± 0.24^d^	28.19 ± 0.71^c^	37.11 ± 0.56^b^	47.89 ± 0.92^a^
	90	20.38 ± 0.35^e^	27.08 ± 0.63^c^	36.48 ± 0.39^b^	.26 ± 0.72^a^

Within the rows and columns of a parameter, means denoted by a different letter are statistically different (*P* < 0.05)

PBMF: Parent Buffalo Milk Fat

LMPF-25: Low Melting Point Fraction of Buffalo Milk Fat Obtained at 25 °C

LMPF-15: Low Melting Point Fraction of Buffalo Milk Fat Obtained at 15 °C

LMPF-10: Low Melting Point Fraction of Buffalo Milk Fat Obtained at 10 °C

### α-Tocopherol and vitamin a

Table 5 presents the concentrations of tocopherol and vitamin A in milk fat and its low melting point fractions. Fractionation temperature significantly influenced the concentrations of α-Tocopherol and vitamin A. LMPF-25, LMPF-15 and LMPF-10 had higher amounts of α-Tocopherol and vitamin A as compared to unmodified milk fat. Concentrations of vitamin A in LMPF-25, LMPF-15 and LMPF-10 were 56, 142 and 206 μg/100 g higher than PBMF. Magnitude of α-tocopherol in LMPF-25, LMPF-15 and LMPF-10 were 9, 33 and 64 mg/100 g higher than PBMF. Higher amounts of α-tocopherol and vitamin A in low melting point fractions of milk fat can be connected to their association with low point fractions. This was also evidenced from the concentrations of unsaponifiable matter in LMPF-25, LMPF-15 and LMPF-10 as compared to PBMF. Vitamins belong to the unsaponifiable fraction of fats and oils [63]. Effect of storage duration on α-tocopherol and vitamin A was found non-significant till 45 days. Estimation of α-tocopherol and vitamin A after 90 days of storage, indicated a significant effect on content of storage on α-tocopherol and vitamin A. After 90 days of storage, decrease in content of α-tocopherol in PBMF, LMPF-25, LMPF-15 and LMPF-10 were 17, 19, 24 and 22 mg/100 g. After 90 days of storage, decrease in content of vitamin A in PBMF, LMPF-25, LMPF-15 and LMPF-10 were 81, 49, 43 and 45 μg/100 g. Previous studies conducted by Andino and Daniel [64] revealed that during 4 weeks of refrigerated storage, vitamins (α-tocopherol) concentration in plain yogurt decrease promptly.

**Table 5:** Vitamin A and tocopherol content of low melting point fractions of buffalo milk fat

Vitamin	Storage Days	PBMF	LMPF-25	LMPF-15	LMPF-10
Vitamin A μg/100 g	0	512 ± 0.22^f^	568 ± 0.88^e^	654 ± 0.65^b^	718 ± 0.49^a^
	45	508 ± 0.39^f^	560 ± 1.36^e^	644 ± 0.32^b^	713 ± 0.78^a^
	90	431 ± 1.19^g^	519 ± 0.77^f^	611 ± 0.91^d^	673 ± 1.44^c^
α-Tocopherol mg/g	0	133 ± 0.29^d^	141 ± 0.18^c^	166 ± 0.25^b^	197 ± 0.37^a^
	45	131 ± 0.17^d^	137 ± 0.42^d^	161 ± 0.39^b^	192 ± 0.66^a^
	90	116 ± 0.35^f^	122 ± 0.12^e^	142 ± 0.59^c^	175 ± 0.19^b^

In one row, if means are expressed with a different letter, these are statistically significant (*P* < 0.05)

PBMF: Parent Buffalo Milk Fat

LMPF-25: Low Melting Point Fraction of Buffalo Milk Fat Obtained at 25 °C

LMPF-15: Low Melting Point Fraction of Buffalo Milk Fat Obtained at 15 °C

LMPF-10: Low Melting Point Fraction of Buffalo Milk Fat Obtained at 10 °C

### Lipid oxidation

Findings on lipid oxidation of low melting point fractions of buffalo milk fat are shown in Table 6. Free fatty acids, peroxide value, anisidine value, iodine value and conjugated dienes were used as indicators of lipid oxidation during the storage of 90 days. Free fatty acids of all the three low melting fractions and unmodified milk fat increased slowly and steadily throughout the storage period (*p* > 0.05). Estimation of free fatty acids after the 90 days of storage duration, no significant difference was found in content of free fatty acids in unmodified milk fat and low melting point fractions. In oils and fats, free fatty acids are generated due to the hydrolytic activities of lipases, moisture, temperature and metal ion contamination etc. [65]. In this investigation, moisture content of PBMF, LMPF-25, LMPF-15 and LMPF-10 were 0.18, 0.16, 0.19 and 0.18%, respectively. Rise of free fatty acids in unmodified milk fat and low melting point fractions may be due to the presence of moisture and ambient storage (25-30 °C). During the course of 3-months storage, free fatty acids in butter oil with modified profile increased [57]. From the processing ability, price and storage stability, fatty acids are important criteria, they may lead to higher process loss and development of bad flavors in fats and oils [66]. Free fatty acids have also been implicated in the catalysis of auto-oxidation in fats and oils during the storage [67]. Among the classical methods of lipid oxidation measurement, peroxide value is frequently used to determine and forecast the oxidative/ storage stability of oils and fats [68]. Peroxide value of freshly harvested low melting point fractions was not different from PBMF. However, storage temperature affected the peroxide value in all the samples of low melting fractions and PBMF. In all the samples of low melting fractions and PBMF, peroxide value remains unchanged up to 45 days of storage. Testing of peroxide value in 90 days old samples showed that peroxide value of PBMF, LMPF-25, LMPF-15 and LMPF-10 was 0.54, 0.98, 1.46 and 2.22 (MeqO_2_/kg). According to standards of European Union, allowable limit of peroxide value of oils and fats is 10 (MeqO_2_/kg). LMPF-10 has about 33% more C_18:2_ than PBMF, which may be the reason of relatively higher peroxide value in LMPF-10. Reynhout [69] described that relative oxidation rate of C_18:2_ was more than times higher than C_18:1_. An inverse relationship was found between peroxide value and iodine value, samples of low melting point fractions having more peroxide value showed lower iodine value. Anwar et al. [70] established a connection between auto-oxidation and peroxide value, samples having more peroxide value had lower peroxide value. The concentration of secondary oxidation products progressively increased in all the low melting point fractions and PBMF. LMPF-10 yielded the highest concentration of secondary oxidation products. A linear correlation of anisidine value and peroxide value was established and observed that fats having higher peroxide value possessed greater anisidine value. Anisidine value measures the concentration of aldehydes in fats and oils; higher levels of aldehydes indicate higher anisidine value with poor keeping quality. The concentrations of conjugated dienes measured as specific extinction at 232 nm. Periodical analysis of low melting point fractions subjected to ambient storage practice revealed a typical fashion of increase in the concentration of conjugated dienes and trienes. After 90-days of storage, the initial value of conjugated dienes LMPF-10 was 0.39.

**Table 6:** Lipid oxidation in low melting point fractions of buffalo milk fat

Parameters	Storage Days	PBMF	LMPF-25	LMPF-15	LMPF-10
Free Fatty Acids	0	0.11 ± 0.01^b^	0.10 ± 0.02^b^	0.11 ± 0.01^b^	0.11 ± 0.02^b^
	45	0.12 ± 0.02^b^	0.11 ± 0.03^b^	0.12 ± 0.02^b^	0.12 ± 0.01^b^
	90	0.15 ± 0.01^a^	0.15 ± 0.02^a^	0.14 ± 0.03^a^	0.14 ± 0.01^a^
Peroxide Value (MeqO_2_/kg)	0	0.25 ± 0.02^d^	0.26 ± 0.03^d^	0.25 ± 0.02^d^	0.28 ± 0.03^d^
	45	0.32 ± 0.04^d^	0.37 ± 0.03^d^	0.35 ± 0.07^d^	0.38 ± 0.05^d^
	90	0.54 ± 0.09^c^	0.98 ± 0.05^b^	1.26 ± 0.13^a^	1.46 ± 0.08^a^
Iodine Value Cg/100 g	0	35.56 ± 0.55^e^	39.11 ± 1.44^d^	42.78 ± 0.34^c^	47.89 ± 0.52^a^
	45	35.13 ± 0.42^e^	39.03 ± 0.45^d^	41.11 ± 0.14^d^	46.72 ± 0.35^a^
	90	34.73 ± 0.15^e^	38.94 ± 0.25^d^	40.58 ± 0.07^d^	44.55 ± 0.21^b^
Anisidine Value	0	4.59 ± 0.23^i^	4.75 ± 0.34^i^	4.78 ± 0.13^i^	4.75 ± 0.17^i^
	45	6.69 ± 0.35^h^	11.98 ± 0.76^f^	13.58 ± 0.14^e^	18.99 ± 0.37^b^
	90	10.73 ± 0.15^g^	18.75 ± 0.81^d^	22.63 ± 0.22^c^	27.85 ± 0.61^a^
Conjugated Dienes (^1%^ ε1CM [**λ**232])	0	0.39 ± 0.02^g^	0.39 ± 0.02^g^	0.39 ± 0.02^g^	0.39 ± 0.02^g^
	45	1.88 ± 0.11^f^	2.05 ± 0.15^e^	2.81 ± 0.03^d^	3.25 ± 0.12^c^
	90	3.19 ± 0.27^c^	3.35 ± 0.29^c^	4.46 ± 0.14^b^	5.75 ± 0.11^a^

In the rows and column of one parameter, if means are expressed with a different letter, these are statistically significant (*p* < 0.05)

PBMF: Parent Buffalo Milk Fat

LMPF-25: Low Melting Point Fraction of Buffalo Milk Fat Obtained at 25 °C

LMPF-15: Low Melting Point Fraction of Buffalo Milk Fat Obtained at 15 °C

LMPF-10: Low Melting Point Fraction of Buffalo Milk Fat Obtained at 10 °C

## Conclusion

Low melting point fractions of buffalo milk fat had higher amount of unsaturated fatty acids and conjugated linoleic acids than unmodified milk fat. Triglycerides from C_44_ to C_54_ were intensified in the low melting point factions. Low melting point fractions harvested at 15 and 10oC had stronger free radical scavenging capacity than unmodified milk fat. α-tocopherol and vitamin A were also intensified in low melting point fractions. Up to 45 days, peroxide value, anisidine value, iodine value and conjugated dienes in all the three low melting point fractions were not different from unmodified milk fat. Low melting point fractions can be extracted from milk fat for the formulation of functional foods and industrial applications.
